# Rapid oral bacteria detection based on real-time PCR for the forensic identification of saliva

**DOI:** 10.1038/s41598-018-29264-2

**Published:** 2018-07-18

**Authors:** Ju Yeon Jung, Hyun Kyu Yoon, Sanghyun An, Jee Won Lee, Eu-Ree Ahn, Yeon-Ji Kim, Hyun-Chul Park, Kyungmyung Lee, Jung Ho Hwang, Si-Keun Lim

**Affiliations:** 1 Forensic DNA Division, National Forensic Service, 10, Ipchun-ro, Wonju-si, Gangwon-do, 26460 Republic of Korea; 2 JS Biotech, Business Incubation Center, Kyungbok University, 425 Kyungbokdae-ro, Jinjeop-eup, Namyangju-si, Gyeonggi-do, 12051 Republic of Korea; 3 DNA Analysis Division, Seoul Institute, National Forensic Service, 139, Jiyang-ro, Yangcheon-gu, Seoul 08036 Republic of Korea

## Abstract

This study developed a new method for forensic saliva identification using three oral bacteria, *Streptococcus salivarius*, *Streptococcus sanguinis*, and *Neisseria subflava*, combined with a real-time polymerase chain reaction (RT-PCR) system we called OB mRT-PCR. Analytical sensitivity results showed that the target bacteria were amplified at 10^2^–10^7^ copies/reaction, and analytical specificity was assessed using 24 other viruses, bacteria, and protozoa. To evaluate the OB mRT-PCR kit for forensic applications, saliva from 140 Korean individuals was tested, and at least two target bacteria were detected in all the samples. Additional studies on non-saliva samples demonstrated the specificity of the kit. Comparison of the kit with two conventional saliva test methods, the SALIgAE and RSID-Saliva assays, indicated that it was more sensitive and applicable to saliva samples in long-term storage (up to 14 weeks). Additionally, through amplification of mock forensic items and old DNA samples (isolated without lysis of the bacterial cells, regardless of their Gram-positivity), we found that the kit was applicable to not only saliva swabs, but also DNA samples. We suggest that this simple RT-PCR-based experimental method is feasible for rapid on-site analysis, and we expect this kit to be useful for saliva detection in old forensic DNA samples.

## Introduction

The identification of saliva from stains on evidence is an important procedure in forensic investigations, particularly in those related to sex crimes. In addition, saliva detection on blood stains allows distinguishing expectorated blood spatter, which may aid complete understanding of incidents in violence, robbery, and homicide cases^1,2^. The detection of amylase has been generally used for testing of presumptive saliva, because amylase exists at particularly high levels in it. However, amylase is also found in other body fluids, including blood, urine, semen, and vaginal secretions^3–5^. Additionally, protein-based methods for identification of body fluids have varying degrees of sensitivity according to the inter- and intra-variability of the protein levels^6,7^. Thus, different strategies, using messenger RNA, microRNA, and methylation, capable of detecting the presence of saliva have been studied^6–10^. However, these methods are labour-intensive and time-consuming because RNA handling requires particular care to maintain its stability against ubiquitously present ribonucleases, and methylation studies requires a bisulfite-converting process on the DNA^10^. Detection of oral bacterial DNA based on polymerase chain reaction (PCR) has been studied as a strategy and reported to be simple and sensitive^1,11–13^. Additionally, the study of microbial patterns through sequencing of body fluids has been reported recently^14^. In this study, we developed an oral bacteria multiplex real-time PCR kit, named OB mRT-PCR, as a new forensic saliva identification tool. Real-time PCR offers a number of advantages over conventional PCR, including high sensitivity, improved accuracy, and evaluation of data without post-PCR detection procedures^15^. There are numerous bacterial species and phylotypes in the oral cavity^16^, and the majority of oral bacteria are unique to this habitat^1,12,17,18^. In addition, the environment (eating and drinking habits, tobacco use, age, and chronic nail-biting habits) can change the complex community of oral bacteria^19–23^. For these reasons, one or two oral bacteria may not be enough to determine the presence of saliva. Therefore, we developed the OB mRT-PCR kit using markers for three oral bacteria, *Streptococcus salivarius*, *Streptococcus sanguinis*, and *Neisseria subflava*. The targeted oral bacteria were selected from the literature and experiments on bacterial identification from saliva cultures using a MicroSeq. 500 16 S rRNA-based bacterial ID system^24,25^. According to previous reports, *S. salivarius* is a prominent member of the oral microbiota^26^, *S. sanguinis* is the most abundant species found in oral biofilms^27^, and *N. subflava* comprises part of the normal flora of the oral cavity and respiratory tract of humans^28^. We evaluated whether the OB mRT-PCR kit could provide consistent results and little variability when used on 140 Korean individuals. In the specificity study, blood, urine, vaginal fluids, semen, faeces, and skin samples were examined. The sensitivity of the kit was assessed by comparing it with conventional presumptive and confirmatory saliva testing methods based on amylase, the SALIgAE and RSID-Saliva assays, using saliva subjected to long-term storage (up to 14 weeks). In addition, mock forensic items that had contacted human mouths (cigarettes, straws, mugs, paper cups, forks, and bite marks on corncobs) were tested to evaluate the forensic potential of the kit. The evaluation was mainly performed by direct RT-PCR (without a DNA isolation step)^29,30^ using swabs as templates for rapid detection. Furthermore, we used old DNA samples obtained from real crime scenes as templates. The DNA samples were isolated without additional treatment to lyse microbial cells and had been stored for up to 13 years. Our data showed that the OB mRT-PCR kit was useful as a saliva identification tool and could be applicable not only to swabs, but also to isolated DNA, even that isolated without any particular Gram-positive bacterial cell lysing process.

## Results

### Analytical performance

**Figure 1:**
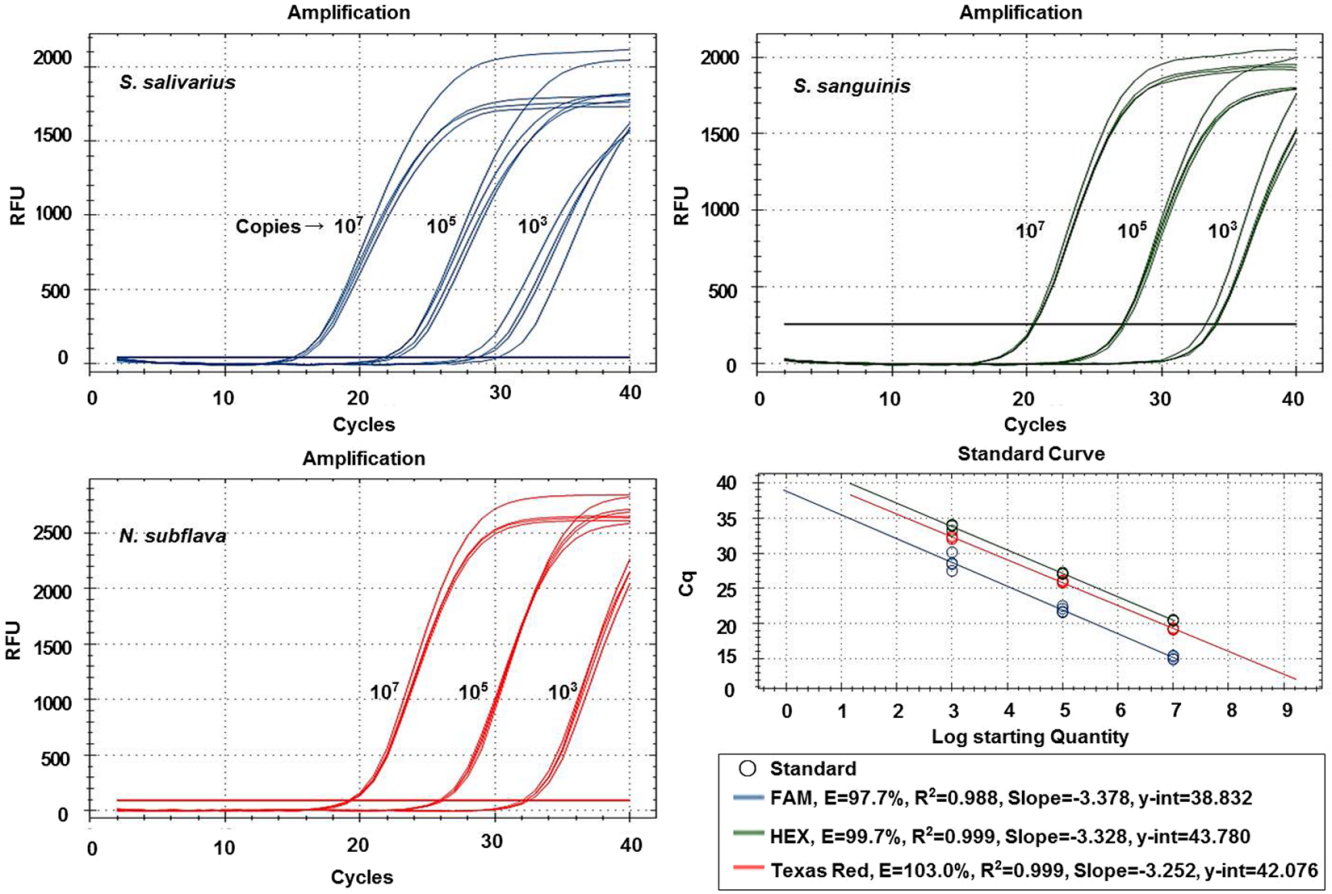
Oral bacteria real-time PCR reaction performed using synthetic genes in the amount of 10^3^, 10^5^, and 10^7^ copies/reaction corresponding to low, intermediate, and high concentrations, respectively. The data were analysed from cycle 2 to 40.

**Figure 2:**
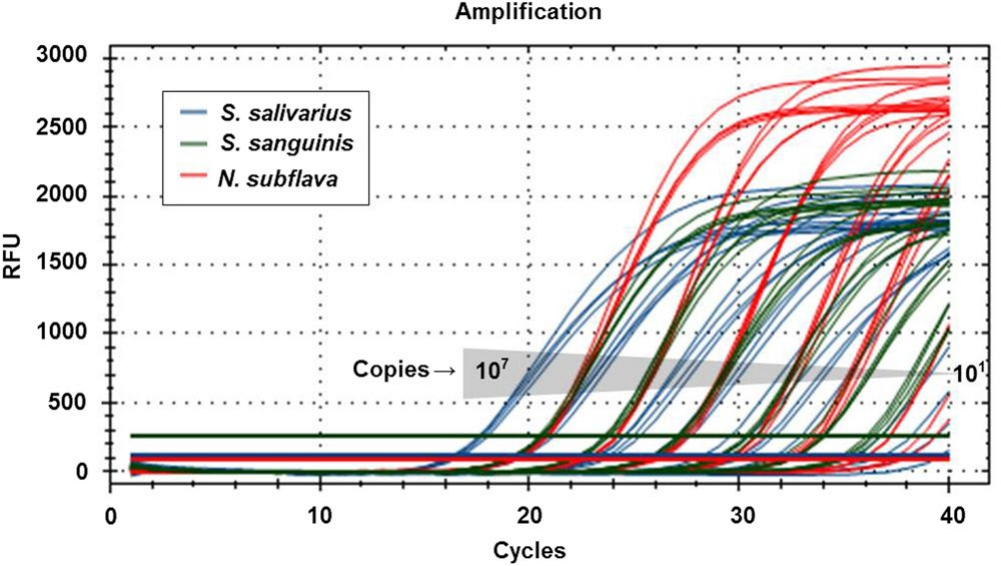
Measurement range test results using synthetic genes to confirm analytical sensitivity. All three bacteria species were detected at 10^2^~10^7^ copies/reaction.

For the evaluation of the analytical performance of the OB mRT-PCR kit, synthetic genes prepared at various concentrations were used. The linearity of the multiplex real-time PCR (mRT-PCR) conditions was measured using synthetic genes in the amount of 10^3^, 10^5^, and 10^7^ copies/reaction, corresponding to low, intermediate, and high concentrations, respectively (*S. salivarius* R^2^ = 0.988, efficiency = 97.7%, *S. sanguinis* R^2^ = 0.999, efficiency = 99.7%, and *N. subflava* R^2^ = 0.999, efficiency = 103.0%; Fig. 1). To confirm the analytical sensitivity and the measurement range, synthetic genes were used at 10^1–^10^7^ copies/reaction and analysed four times for each dilution. The experimental results showed that all three oral bacteria were amplified when used at 10^2^–10^7^ copies/reaction (Fig. 2). The analytical specificity was evaluated using 24 other viruses, bacteria, and protozoa (Table 1), none of which was amplified.

**Table 1:** Analytical specificity test panel.

Species	Strain	Species	Strain
*Entamoeba histolytica*	DS4-868	*Campylobacter jejuni*	Clinical isolate
*Giardia lamblia*	H3	*Campylobacter coli*	Clinical isolate
*Cryptosporidium parvum*	Iowa	*Vibrio cholerae*	Z133; non-toxigenic
*Pleisomonas shigelloides*	Z130	*Clostridium difficile*	NAP1
*Cyclospora cayetanensis*	recombinant	*Clostridium perfringens*	NCCP 10347
*Escherichia coli*	EDL933; O157	*Candida parapsilosis*	KCTC 7214
*Escherichia coli*	92.0147; EAEC	*Streptococcus sobrinus*	KCTC 3288
*Escherichia coli*	ETEC; ST + , LT+	*Streptococcus gordonii*	KCTC 3286
*Escherichia coli*	7.1493; O84:H28; EPEC	Influenza A H1N1 virus	A/NewCaledonia/20/99
*Shigella sonnei*	Z004	Influenza A H1N1 virus	A/Brisbane/59/07
*Salmonella enterica typhimurium*	Z005	Influenza B virus	B/Florida/02/06
*Yersinia enterocolitica*	Clinical isolate	Influenza B virus	B/Malaysia/2506/04

### Evaluation of the OB mRT-PCR kit

**Figure 3:**
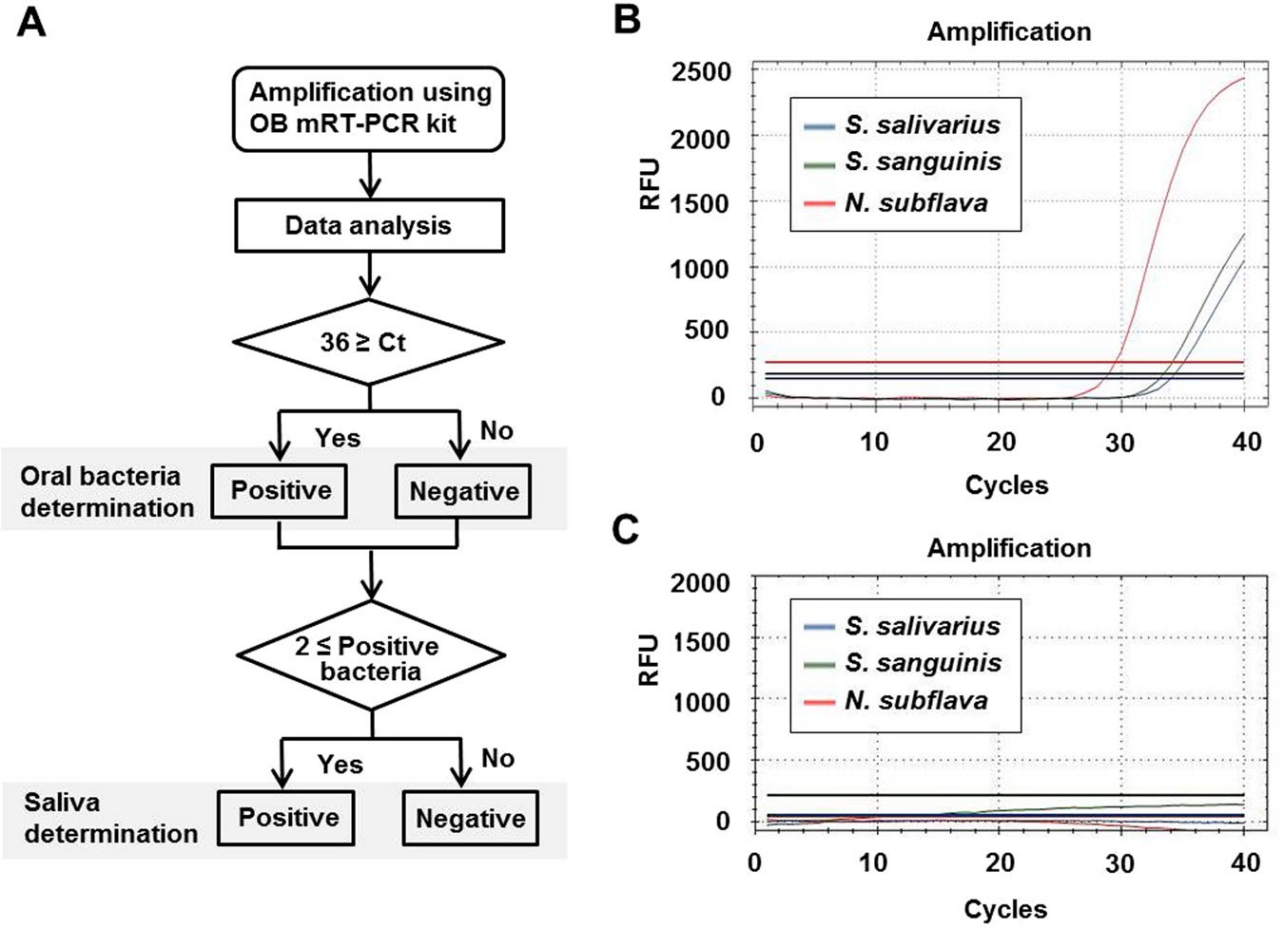
Criteria for determination of the presence of saliva (**A**) and two examples of RT-PCR results obtained from a saliva sample (**B**) and distilled water (**C**); (**C**) is the negative control, with no DNA) analysed using the OB mRT-PCR kit.

**Figure 4:**
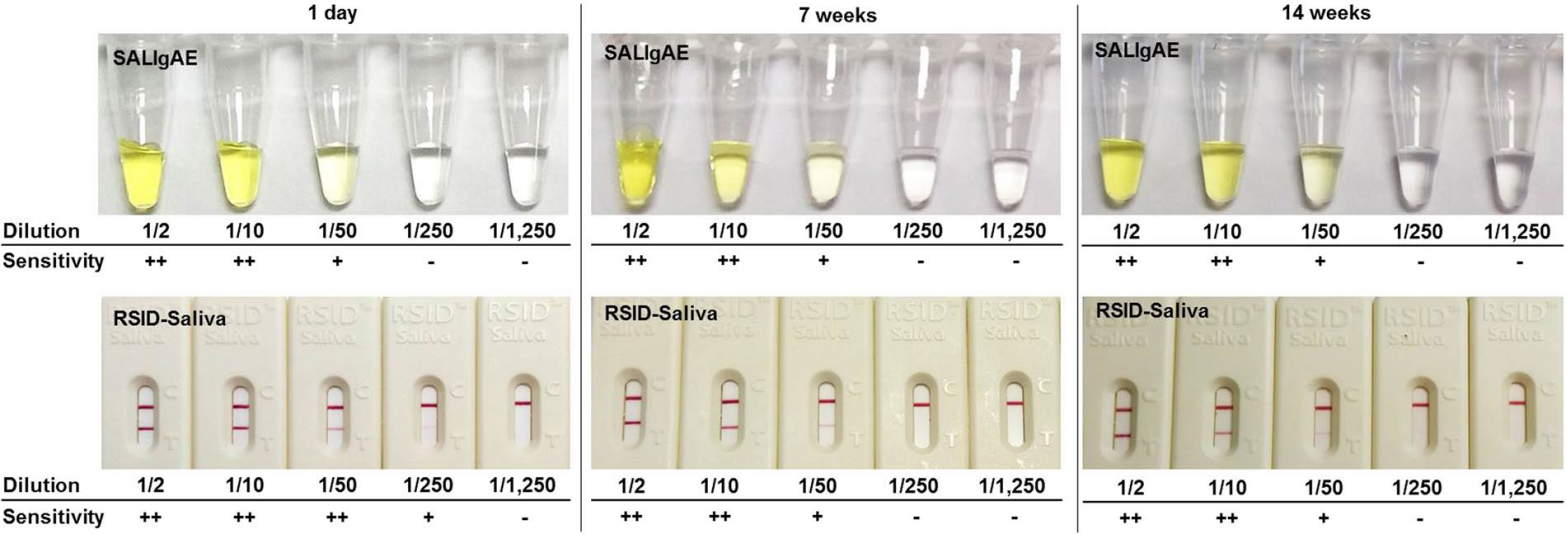
Sensitivity study of conventional saliva detection methods, the SALIgAE and RSID-Saliva assays, using diluted saliva.

The OB mRT-PCR kit was evaluated upon amplification of 140 saliva samples from different individuals: this analysis is particularly important because the oral bacterial community differs among individuals. The saliva samples were collected using swabs, and a piece (2-mm × 2-mm) of the swab was directly used as a template for the test. The results showed that at least two oral bacteria species were detected in all saliva samples (Table 2). This supports the ‘positive’ result criteria of the OB mRT-PCR kit indicating that at least two bacterial species should be detected to determine presence of saliva (Fig. 3). The three bacterial species were detected by the kit in 91.4% samples. Of these, *S. salivarius* was found most abundantly in saliva samples and was not detected in only one sample. *S. sanguinis* was not detected in four, and *N. subflava* was not detected in seven of the 140 samples. The estimated mean Ct values of these oral bacteria were 24.1 ± 1.8 (*S. salivarius*, n = 139), 27.0 ± 1.5 (*S. sanguinis*, n = 136), and 23.8 ± 2.1 (*N. subflava*, n = 133). To observe the influence of tooth brushing, 24 saliva samples (in which all oral bacteria were detected) were additionally collected 10 min after brushing and tested. Tooth brushing had little effect on the detection of the oral bacteria (Table 3); although the Ct values increased slightly, all were within the positive range (Ct values should be below 36 to be reliable). In the specificity study, blood, urine, vaginal fluid, semen, faeces, and skin samples were tested and were all identified as negative except one faeces sample (Table 4), which was positive for *S. salivarius* with an average Ct value of 30.7. In the sensitivity study of the OB mRT-PCR kit, we diluted whole saliva 1/2, 1/10, 1/50, 1/250, and 1/1,250 using distilled water and stored the samples at 4 °C for 1 day, 7 weeks, or 14 weeks. One microliter of diluted saliva was used with the OB mRT-PCR kit, SALIgAE assay, and RSID-Saliva assay, as described herein. The results showed that the OB mRT-PCR kit and RSID^TM^-Saliva assay were more sensitive than the SALIgAE assay was when using 1-day old samples (Fig. 4 and Table 5). At 7 weeks, the sensitivity of the RSID-Saliva assay slightly decreased (Fig. 4). Interestingly, oral bacteria detection was slightly higher after storage for 7 weeks than after storage for 1 day. The OB mRT-PCR kit was the only able to detect the saliva in all 1/250 and some 1/1,250 diluted saliva samples.

**Table 2:** Number of positive amplified oral bacteria (Ct values below 36) in 140 Korean saliva samples which were collected at least two hours after tooth brushing.

Oral bacteria	Sample (N)	Ratio (%)
*S. salivarius* + *S. sanguinis* + *N*. *subflava*	128	91.4
*S. salivarius* + *S. sanguinis*	7	5
*S. salivarius* + *N. subflava*	4	2.9
*S. sanguinis* + *N. subflava*	1	0.7
Total	140	100

**Table 3:** Changes in Ct values of 24 saliva samples according to tooth brushing.

Oral bacteria	Collected at least 2 hours after TB		Collected previous 10 min after TB	
	Mean	Std	Mean	Std
*S. salivarius*	25.5	3.4	26.0	3.6
*S. sanguinis*	26.9	2.9	29.3	2.8
*N. subflava*	22.5	4.0	24.6	3.4

TB: tooth brushing; Std: standard deviation.

**Table 4:** Specificity study of the OB mRT-PCR kit with various body fluid samples.

Oral bacteria	Blood	Urine	Vaginal fluids	Semen	Faeces	Skin
*S. salivarius*	N (10)	N (10)	N (10)	N (5)	N (2), P (1)	N (5)
*S. sanguinis*	N (10)	N (10)	N (10)	N (5)	N (2)	N (5)
*N. subflava*	N (10)	N (10)	N (10)	N (5)	N (2)	N (5)

P: positive, N: negative. The number in brackets indicates the numbers of negative (N) or positive (P) samples.

**Table 5:** Sensitivity study of the OB mRT-PCR kit using diluted saliva.

Time	Dilution	Sample A			Sample B			Sample C			Final conclusion
		*S.Sal*	*S.San*	*N.Sub*	*S.Sal*	*S.San*	*N.Sub*	*S.Sal*	*S.San*	*N.Sub*	
1 day	1/2	P	P	P	P	P	P	P	P	P	P (3)
	1/10	P	P	P	P	P	P	P	P	P	P (3)
	1/50	P	P	P	P	P	P	P	P	P	P (3)
	1/250	P	P	P	P	N	P	P	N	P	P (3)
	1/1,250	N	N	P	N	N	P	N	N	P	P (1), N (2)
7 weeks	1/2	P	P	P	P	P	P	P	P	P	P (3)
	1/10	P	P	P	P	P	P	P	P	P	P (3)
	1/50	P	P	P	P	P	P	P	P	P	P (3)
	1/250	P	P	P	P	P	P	P	P	P	P (3)
	1/1,250	N	N	P	N	N	P	N	N	P	P (1), N (2)
14 weeks	1/2	P	P	P	P	P	P	P	P	P	P (3)
	1/10	P	P	P	P	P	P	P	P	P	P (3)
	1/50	P	P	P	P	P	P	P	P	P	P (3)
	1/250	P	P	P	P	P	P	P	P	P	P (3)
	1/1,250	N	N	P	N	N	P	P	N	P	P (1), N (2)

*S. Sal: S. salivarius, S. San: S. sanguinis, N. Sub: N. subflava*, P: positive, N: negative. The number in brackets indicates the numbers of negative (N) or positive (P) samples.

### Forensic application

A total of 40 mock forensic items were obtained, including cigarettes, straws, mugs, paper cups, forks, and bite marks on corncobs. Pieces (2-mm × 2-mm) of the cigarettes and swabs used to wipe the mock forensic items were used directly as templates. All target bacteria were positively detected in 82.5% of samples (33/40 samples), and two target bacteria in the other 17.5% (7/40 samples). The calculated mean Ct values of the total items were 30.9 ± 2.1 (*S. salivarius*, n = 40), 32.4 ± 1.8 (*S. sanguinis*, n = 35), and 29.3 ± 1.9 (*N. subflava*, n = 38). Comparing the RT-PCR results from the mock forensic samples with those from the 140 Korean saliva samples, the detection rate using the mock forensic samples was lower and mean Ct values were higher than those measured using the 140 saliva samples (Table 6). However, since two or more bacteria were detected in all samples, the samples were all considered positive for ‘saliva presence’. To verify that the OB mRT-PCR kit can be applied to DNA samples, we amplified various DNA samples as described in Table 7. Ten DNA samples (isolated from evidence containing saliva) were stored between 6 months and 13 years, and the total DNA concentrations ranged from 0.13 ng/µL to 4.76 ng/µL (Table 7). As negative controls, six DNA samples isolated from other body fluids (blood, vaginal fluid, and semen) were amplified simultaneously. We found that one vaginal sample tested positive for *S. salivarius*. However, the detection of one bacterial does not meet the criteria for ‘saliva presence’. All saliva DNA samples except one (cigarette butt stored for 7 years) were determined to be positive by the OB mRT-PCR kit. All target bacteria were positively detected in 40% of samples (4/10 samples), and two target bacteria were positively detected in 50% of the samples (5/10 samples). The mean Ct values of the DNA samples containing saliva were 31.6 ± 3.3 (*S. salivarius*, n = 7), 33.5 ± 1.5 (*S. sanguinis*, n = 8), and 32.9 ± 2.7 (*N. subflava*, n = 8). The detection rate of target bacteria when using old DNA was lower than that when using the mock forensic samples. Nevertheless, the data obtained demonstrate that the OB mRT-PCR kit can be used with old DNA samples. The DNA samples used in our analysis were isolated using a QIAmp DNA micro kit (Qiagen, Hilden, Germany), and no additional treatment was performed to lyse bacterial cell walls. This indicates that the OB mRT-PCR kit is a useful tool for the detection of saliva in old DNA samples that have not been tested for saliva, even if the samples were isolated without specific treatment to lyse bacterial cells. In a previous report, the same kit has been used for the analysis of the microbial diversity of oral saliva^31^. Since the QIAmp DNA micro kit is not designed specifically for bacterial cells, additional treatments might be required for the complete lysis of Gram-positive bacterial cells^32^. However, two of the bacteria used as markers in our study were Gram-positive, and though other kits might be found more convenient/efficient in this regard, the QIAmp DNA micro kit did efficiently allow their amplification.

**Table 6:** Results of amplified oral bacteria using the OB mRT-PCR kit in mock forensic samples.

Samples	No. of samples	*S. salivarius*			*S. sanguinis*			*N. subflava*		
		No. of Positive	Ct		No. of Positive	Ct		No. of Positive	Ct	
			Mean	Std		Mean	Std		Mean	Std
Cigarette	10	10	31.0	1.6	10	30.4	2.2	9	32.4	2.6
Straw	10	10	32.7	1.8	9	34.1	1.7	10	28.7	2.2
Mug cup	5	5	31.8	1.1	4	34.4	1.2	5	29.4	1.9
Paper cup	5	5	31.8	2.2	5	32.6	2.4	4	28.8	1.4
Fork	5	5	31.0	2.1	3	32.6	3.7	5	29.8	2.5
Bite mark on corncobs	5	5	26.8	1.1	4	30.0	1.7	5	26.6	2.7

No. of positive indicates Ct values under^36^. Std: standard deviation.

**Table 7:** Results of amplified oral bacteria using the OB mRT-PCR kit in forensic DNA samples.

No.	Sample source (evidence)	Storage	Case type	Conc. of total DNA	*S. salivarius*			*S. sanguinis*			*N. subflava*			Final conclusion
					P/N	Ct		P/N	Ct		P/N	**Ct**		
						M	Std		M	Std		M	Std	
1	Saliva on soil	13 years	Homicide	1.33	P	26.9	0.1	P	31.9	0.3	P	27.7	0.2	P
2	Saliva on wastepaper	11 years	Rape	0.53	P	28.4	0.0	P	34.2	0.8	P	34.5	0.2	P
3	Cigarette butt	11 years	Thief	0.33	P	29.8	0.2	P	32.2	0.6	N			P
4	Saliva on box	8 years	Thief	0.59	P	35.9	0.1	P	35.5	0.3	P	30.6	0.3	P
5	Cigarette butt	7 years	Thief	0.14	N			N			P	35.7	0.4	N
6	Bite mark on food	3 years	Thief	4.76	N			P	33.5	0.3	P	35.1	0.3	P
7	Saliva on glass	1 year	Fraud	0.85	P	32.9	0.2	N			P	31.7	0.3	P
8	Saliva on window	1 year	Thief	0.13	P	32.3	0.3	P	33.2	0.1	P	34.3	0.2	P
9	Tooth brush	8 months	Rape	2.64	P	34.7	0.4	P	31.9	0.2	N			P
10	Cigarette butt	6 months	Rape	0.25	N			P	35.4	0.1	P	33.4	0.2	P
11	Blood on soli	11 years	Homicide	0.93	N			N			N			N
12	Blood on gloves	8 years	Homicide	0.97	N			N			N			N
13	Vaginal fluid	13 years	Homicide	5.83	P	32.7	0.2	N			N			N
14	Vaginal fluid	9 years	Rape	0.13	N			N			N			N
15	Semen	13 years	Rape	3.46	N			N			N			N
16	Semen	7 years	Rape	1.8	N			N			N			N

Conc.: concentration, P: positive, N: negative, M: mean, Std: standard deviation.

In the National Forensic Service (NFS) of the Republic of Korea, most of the old forensic DNA samples have been isolated by conventional human genomic DNA preparation methods without additional treatments to lyse microbial cell walls, because the intention of the isolation was human DNA profiling. Our kit can potentially be used with such samples. Additionally, even on DNA samples stored up to 13 years, oral bacteria were detectable in as little as 0.13 ng of total DNA. The amount of total DNA is not the amount of bacterial DNA: most of the DNA is indeed human genomic DNA and the amount of bacterial DNA is very small. Therefore, our data demonstrate that large amounts of DNA are not required to identify saliva using the OB mRT-PCR kit, and that this kit can be successfully used with old DNA (stored for up to 13 years).

## Discussion

Various approaches for saliva identification have been studied^33^, and the use of oral bacteria detection-based PCR has also been reported^11–13^. In this paper, we developed an oral bacteria multiplex RT-PCR kit (named OB mRT-PCR) for forensic saliva identification using three target oral bacteria, *S. salivarius*, *S. sanguinis*, and *N. subflava* as markers. Due to environmental factors, the types and amounts of oral bacteria vary among individuals; the OB mRT-PCR kit will improve the accuracy of saliva identification by simultaneously analysing three species of bacteria as targets. The kit was evaluated by analysing 140 Korean saliva samples; the results showed that two or more oral bacterial species (targeted by the kit) were detected in all samples. In a specificity study of various body fluid samples, *S. salivarius* was detected in one faeces sample, consistently with the results of a previous report^34^. Additionally, *S. salivarius* was detected in one forensic DNA sample isolated from vaginal fluid. However, the identification of only one bacterial species does not indicate presence of saliva according to the criteria we indicated. Because bacteria are associated with some diseases^35–38^, their presence in other tissues, in some instances, cannot be excluded. Only one bacterial target was detected in the faeces sample and vaginal DNA sample; thus, these samples were classified as negative. For accurate determinations, each laboratory may need an internal validation study to establish the criteria for positive determination. The consideration of the donor’s clinical information and the analysis of various negative controls (such as no saliva and/or no DNA samples, and swab of experimental space to investigate the environmental bacteria) might help to increase the accuracy of the test.

The high sensitivity of the RSID-Saliva assay has been reported previously^2,39^, but since it is a protein-based method, the sensitivity of this assay decreases if samples are stored for more than seven weeks. On the other hand, the OB mRT-PCR kit is a bacterial DNA-detecting method, which features high sensitivity and a long persistence of sensitivity, and therefore, it is superior when used with forensic samples that have been stored for a long time. In addition, the OB mRT-PCR kit can be used both with mock forensic samples and as well as DNA samples. In recent years, there have been important legal changes in the Republic of Korea, including the extension of the period of prescription for murder cases and sexual crimes. These changes have increased the demand for re-analysis of evidence and/or stored DNA held by the NFS^40^. Furthermore, as Short Tandem Repeat (STR) analysis techniques evolve and become more sensitive and with higher tolerance to inhibitors, the detection rate of STR (autosomal STR and Y chromosome-STR) has increased^41–43^. The importance of accurate identification of the source of the DNA profile has also increased because body fluids can be used as indicators of the sequence of events^44^. The OB mRT-PCR kit has the advantage of being able to identify the presence of saliva in old DNA. Saliva identification by methylation can also be used with old DNA, but relatively high concentrations of DNA are required^45^. Thus, we suggest that the OB mRT-PCR kit is more suitable for identification of saliva in the presence of low amounts of DNA. Considering these results, the OB mRT-PCR kit will be a useful confirmatory tool for the identification of saliva not only from swabs, but also from isolated DNA.

## Conclusions

This study aimed to develop a new saliva identification tool and evaluated its use on samples from Korean individuals, mock forensic samples, and DNA samples. Three species of oral bacteria (*S. salivarius*, *S. sanguinis*, and *N. subflava*) were selected as targets for mRT-PCR. In all 140 Korean individuals, mock forensic samples, and DNA samples (except one DNA sample), DNA from two or more oral bacterial species was successfully amplified with the OB mRT-PCR kit (Ct values below 36). Although *S. salivarius* was found in other samples, only samples containing saliva were determined to be positive according to the criteria for positive identification we defined (detection of two or more of the target bacterial species). The advantage of the OB mRT-PCR kit is that the experimental process is simple and fast through direct RT-PCR, and it can be used to analyse even DNA samples at very small concentration.

## Methods

### Samples

Bacteria used in this study were purchased from the National Culture Collection for Pathogens (NCCP, Osong, Korea) and the Korean Collection for Type Cultures (KCTC, Jeongeup, Korea). Some inactivated bacteria, viruses, and protozoa were purchased from ZeptoMetrix (Buffalo, NY, USA).

For selection of the target bacteria, the expectorate from four healthy volunteers was collected into 50-mL sterile plastic centrifugation tubes. For evaluation of the OB mRT-PCR kit, samples from 140 Korean volunteers (79 males and 61 females) were collected using sterile cotton swabs (Fisher Scientific, Hampton, NH, USA) at least two hours after tooth brushing. Saliva samples from 24 volunteers (12 male and 12 female) were additionally collected 10 min after tooth brushing in order to observe the effects of tooth brushing on bacterial detection. For the specificity study on other body samples, blood (N = 10), urine (N = 10), vaginal fluid (N = 10), semen (N = 5), faeces (N = 3), and skin (N = 5) samples were collected using sterile cotton swabs. Five skin samples were collected by wiping the arm skin with a wet cotton swabs, and all swabs were allowed to dry for 24 h at 25 °C, and were stored at 4 °C until analysis. For a sensitivity comparison between the OB mRT-PCR kit and commercial saliva test kits, whole saliva from three volunteers was additionally collected in 1.5-mL sterile tubes.

A total of 40 mock forensic samples^46–48^ were from 10 cigarettes, 10 straws, 5 mugs, 5 paper cups, 5 forks, and 5 bite marks on corncobs. Cigarettes were cut into 2-mm × 2-mm pieces, and the other mock forensic samples were wiped with swabs soaked in distilled water, dried for 24 h at 25 °C, and then cut into 2-mm × 2-mm pieces. A total of 16 forensic DNA samples were isolated from crime scene evidence that had been analysed by the NFS from 2005 to 2016 for forensic purposes. Ten saliva, 2 semen, 2 blood, and 2 vaginal fluid samples were used. The use of the samples and the analytical procedures involved were approved by the Intuitional Review Board of the National Forensic Service of the Republic of Korea and in accordance with the standards of the Declaration of Helsinki. All volunteers provided written informed consent and all methods were confirmed in accordance with the relevant guidelines and regulations.

### Determination of the target bacteria

Three bacterial species have been reported by many publications to be commonly found in saliva. We determined the targets by considering the results of bacterial identification studies on cultured saliva. Four saliva samples were cultured on blood agar plates (KOMED, Seongnam Korea) supplemented with 1.5% (w/v) pancreatic digest of casein, 0.5% (w/v) pancreatic digest of soybean meal, 5% sheep blood, 0.5% (w/v) sodium chloride (NaCl), and 1.5% (w/v) agar. All cultures were incubated for 18 h in an atmosphere of natural air at 37 °C^49^. Colonies were amplified using the MicroSEQ. 500 Kit bacterial ID system (Perkin-Elmer, Foster City, CA, USA). Amplified 16 S rDNA fragments from the target bacteria were purified with the ExoSAP-IT (Thermo Fisher Scientific, Waltham, MA, USA) treatment and sequenced by the Sanger method. The MicroSEQ ID software v3.0 (Perkin-Elmer) with the AB_Bacteria500Lib_2013 library sets was used on the obtained 16 S rDNA sequences from target unknown bacteria, and the identification process was conducted automatically.

### RT-PCR

In order to extract nucleic acids from cultured bacteria and some inactivated bacteria, viruses, and protozoa, we used a QIAamp DNA mini kit (Qiagen), and a GeneJET viral DNA and RNA purification kit (Thermo Fisher Scientific). Nucleic acids were extracted according to the experimental method and stored at 4 °C until analysis. The following target genes of three oral bacteria were selected for amplification: methionine aminopeptidase (*S. salivarius*), species-specific intergenic spacer region (*S. sanguinis*), and aspartate semialdehyde dehydrogenase (*N. subflava*)^50–52^. The specific sequences were confirmed to have no cross-reaction with other microorganisms by *in silico* analysis. The primers and probes were synthesized by Bioneer (Daejeon, Korea) as described in Table 8. The real-time PCR mixture contained 10 µL of AttoPlex 2X Real-time PCR Master Mix (JS Biotech, Namyangju, Korea), 1 µL of each primer (concentration, 10 µM), 1 µL of each probe (concentration, 5 µM), 1 µL of DNA extracts, and 7 µL of nuclease-free water. The thermal cycle conditions were as follows: 5 min denaturation at 95 °C, 40 cycles of 95 °C for 10 s, and 60 °C for 60 seconds. Real-time PCR was performed using the CFX-96 real-time PCR detection system (Bio-Rad, Hercules, CA, USA), and the cut-off values were set automatically at every run.

**Table 8:** Sequences of the primers and probes for the target genes of the three oral bacteria contained in the OB mRT-PCR kit.

Oral bacteria	Primer/ Probe	Sequence (5′-3′)	Length (bp)	Tm (°C)	Expected size (bp)
*S. salivarius*	Forward	tgaacaagcrgtwgtcggtaac	22	56.3	108
	Reverse	actccgtgtccaaccaaatc	20	55.2	
	Probe	FAM-agtcgtggttacggtgttgttcgt-BHQ1	24	60.6	
*S. sanguinis*	Forward	ggttaatgccgataatgcgatg	22	54.4	108
	Reverse	cggctcatatcgtaaattccaatg	24	54.1	
	Probe	HEX-tgccttgggctatttagtcagcct-BHQ1	24	60.4	
*N. subflava*	Forward	ccaacgatgttgccgaattg	20	55.0	79
	Reverse	tggaagacggatttggtgtaat	22	54.2	
	Probe	TexasRed-ttatcgttacctgtcagggtggcg-BHQ1	24	60.6	

### Performance test

To confirm the amplification sensitivity and linearity, genes containing the amplified sequence of each oral microorganism were synthesized by Bioneer and prepared at a concentration of 10^1^–10^7^ copies/µL. The amplification sensitivity under mRT-PCR conditions was determined four times for each concentration. To confirm the linearity of the amplification conditions, three concentrations (10^3^, 10^5^, and 10^7^) were used. Similarly, the PCR efficiency and the R^2^ values were confirmed by repeating the experiment four times. Specificity experiments used 24 bacteria, viruses, and protozoa to confirm cross-reactivity.

### Evaluation and forensic application of the OB mRT-PCR kit

To evaluate the performance of the OB mRT-PCR kit in 140 Korean individuals, we prepared approximately 2-mm × 2-mm pieces of the top surfaces of swabs. Each piece was directly incubated with 19 µL of the OB mRT-PCR kit reaction mixture on the CFX-96 real-time PCR instrument. Tests were carried out in triplicate and repeated three times. We established two criteria for the positive determination of saliva presence by the OB mRT-PCR kit (Fig. 3a): first, the detected Ct values had to be below 36, and second, more than two oral bacterial species (among the three target bacteria detected by the kit) had to be detected.

The sensitivity was accessed by comparing the OB mRT-PCR kit with two commercial saliva detection kits, the SALIgAE assay kit (Abacus Diagnostics, West Hills, CA, USA) and the RSID-Saliva assay kit (Independent Forensics, Lombard, IL, USA) using three diluted whole human saliva samples. The samples were diluted in distilled water 1/2, 1/10, 1/50, 1/250, and 1/1,250. In order to observe the influence of storage time, the RT-PCR and serology tests were performed after 1 day, 7 weeks, and 14 weeks of storage. One microliter of diluted saliva was used with the OB mRT-PCR kit, SALIgAE assay, and the RSID-Saliva assay. In the SALIgAE assay, 1 µL diluted saliva was added to 30 µL of the SALIgAE solution, and the test results were read after 10 min. In the RSID-Saliva assay, 1 µL of diluted saliva was added to 50 µL of extraction buffer and incubated for 1 h at room temperature, and then mixed with 50 µL running buffer. The test results were read after 10 min. All analyses were carried out in triplicate.

The collected 40 forensic samples were cut into 2-mm × 2-mm pieces, each of which was mixed with 19 µL reaction mixture from the OB mRT-PCR kit. All analyses were carried out in triplicate.

The forensic DNA samples were collected described as Table 7. All DNA samples were isolated using a QIAmp DNA micro kit, and no treatment to lyse bacterial cell walls was performed. One microliter of DNA was used as a template for RT-PCR. DNA samples were quantified in triplicate using a Qubit dsDNA HS Assay Kit (Life Technologies, Darmstadt, Germany), and the Qubit 2.0 Fluorometer (Life Technologies) according to the manufacturer’s instructions. In the calculation of mean Ct values, negative values were excluded.
